# Surface interrogation of advanced electrocatalysts by scanning electrochemical microscopy: fundamentals, progress and perspectives

**DOI:** 10.1039/d5sc04951b

**Published:** 2025-09-08

**Authors:** Rui Yang, Lin Yang, Wenhao Yong, Panpan Li, Min Zhou, Zhaoyu Jin

**Affiliations:** a Institute of Fundamental and Frontier Sciences, University of Electronic Science and Technology of China Chengdu 611731 China zjin@uestc.edu.cn; b College of Materials Science and Engineering, Sichuan University Chengdu 610065 China; c State Key Laboratory of Electroanalytical Chemistry, Changchun Institute of Applied Chemistry, Chinese Academy of Sciences Changchun 130022 China mzhou1982@ciac.ac.cn; d School of Applied Chemistry and Engineering, University of Science and Technology of China Hefei 230026 China

## Abstract

*In situ* quantitative analysis of electrode surfaces, particularly the identification of active sites and reaction intermediates, is essential for elucidating structure–activity relationships and understanding the mechanisms underlying electrocatalytic reactions. Although conventional spectroscopic techniques are widely used to probe catalytic intermediates, they often face limitations in sensitivity, spatial and temporal resolution, and the ability to capture transient phenomena. These constraints hinder the precise quantification of catalytically active sites and the accurate assessment of electrochemical reaction kinetics. The development of surface interrogation scanning electrochemical microscopy (SI-SECM) has enabled the direct and quantitative evaluation of reactive species as well as real-time measurement of reaction kinetics at electrode interfaces. SI-SECM provides valuable insights into the adsorption behavior of intermediates, the complexity of reaction mechanisms, and the characteristics of catalytically active sites. This work introduces the fundamental principles and instrumentation of SI-SECM, with particular attention to its application in key electrocatalytic processes such as hydrogen evolution, oxygen evolution/reduction, ammonia electrosynthesis and carbon dioxide reduction reactions. The article further outlines standard operational protocols for SI-SECM and discusses future directions, including the advancement of high-throughput methodologies, expansion to a broader range of catalytic systems, and progress in multi-field coupling. These developments would collectively position SI-SECM as a versatile platform for mechanistic studies and the rational design of advanced electrocatalysts.

## Abstract 2

This perspective introduces the fundamentals of surface interrogation scanning electrochemical microscopy, highlights recent advances in probing electrocatalytic processes, and discusses future opportunities for multidisciplinary development.

## Graphical abstract

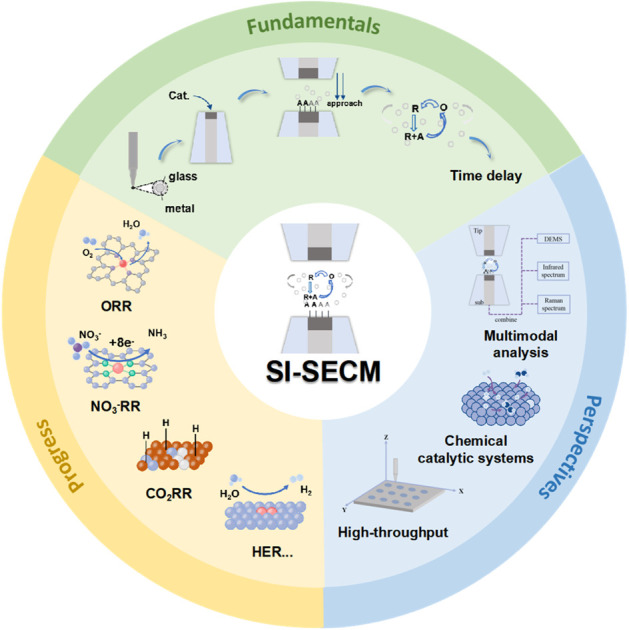

## Introduction

In response to the urgent challenges posed by the global energy crisis and climate change, the development of clean and sustainable energy conversion and storage technologies has become a key focus of scientific research.^1^ Due to its high efficiency and controllability, electrocatalysis plays a vital role in critical reactions such as the hydrogen evolution reaction (HER) and carbon dioxide reduction (CO_2_RR).^2–7^ In this context, *in situ* quantitative analysis of key information on electrode surfaces—such as active sites and reaction intermediates—is essential for elucidating structure–activity relationships and understanding the fundamental mechanisms that govern electrocatalytic processes.^8,9^ Current studies often rely on conventional spectroscopic methods to identify the structure of catalytic intermediates, including X-ray absorption spectroscopy, X-ray photoelectron spectroscopy, X-ray diffraction, Raman spectroscopy, infrared spectroscopy, mass spectrometry, and online inductively coupled plasma mass spectrometry.^10–13^ However, these techniques are inherently limited in terms of sensitivity, spatiotemporal resolution, and their ability to capture dynamic processes, which hampers effective measurement of catalytically active sites and accurate identification of electrochemical reaction kinetics.^14^ Against this backdrop, scanning electrochemical microscopy (SECM) has emerged as a powerful *in situ* characterization tool, capable of providing high-resolution, quantitative insights into electrocatalytic and photoelectrocatalytic processes.

SI-SECM is a specialized operating mode of SECM that combines high sensitivity and temporal resolution. It enables microscale quantitative detection of reaction intermediates and the measurement of reaction kinetics, thereby offering critical information on the behavior of adsorbed species, the complexity of reaction mechanisms, and the nature of active sites on electrode surfaces. In addition to SI-SECM, other quantitative active site probing methods have also been reported, such as CO chemisorption and NO_2_^−^ probing. The CO chemisorption method typically requires a gas-phase environment and is usually performed under *ex situ* conditions, which makes it difficult to capture the information on the truly active sites under electrolyte environments.^15^ The NO_2_^−^ probing method mainly relies on its selective reactivity toward Fe–N_*x*_ active sites in Fe-NC catalysts. For catalysts lacking such sites, the reaction pathways may not be unique, which complicates signal interpretation and limits the general applicability of this method.^16^ In contrast, SI-SECM employs an electrochemical titration strategy, in which controllably generated redox mediators selectively react with active species adsorbed on the electrode surface, thereby directly detecting the active sites involved in the reaction.

SI-SECM was first developed by Bard *et al.* in 2008 as a significant extension of the SECM technique. This technique enables *in situ* detection and quantitative analysis of adsorbed species on electrode surfaces through a unique transient feedback mode. Compared to conventional SECM, SI-SECM requires that the tip and substrate electrodes have matching ultramicroelectrode dimensions and maintain a normalized distance of *L* = *d*/*a* ≤ 0.3 (for example, when the electrode radius *a* = 12.5 μm, the gap *d* is controlled within 1–3 μm), significantly enhancing sensitivity. Moreover, SI-SECM overcomes limitations of traditional electrochemical methods, allowing for precise characterization of surface-adsorbed species under both open-circuit and polarized conditions.^17^ By 2010, Bard *et al.* combined SI-SECM with finite element numerical simulations to investigate the reaction kinetics between oxidized platinum electrode films and various reductants, validating the applicability of Marcus theory within this system.^18^

Since then, the integration of SI-SECM with numerical modeling has enabled *in situ* quantitative analysis of surface oxide reaction kinetics, establishing a new paradigm for studying the reactivity of adsorbed species on electrode surfaces.

The application of the SI-SECM technique in the field of electrocatalysis began in 2015, when Ahn *et al.* first employed it to study CoPi catalysts, successfully determining the surface active site density and the kinetic parameters of the reaction between Co^3+^/Co^4+^ species and water.^19^ That same year, the team further advanced the technique by introducing an external rapid switching device, improving the temporal resolution to the microsecond level, thereby laying the foundation for investigating fast-reacting intermediates.^20^

**Fig. 1:**
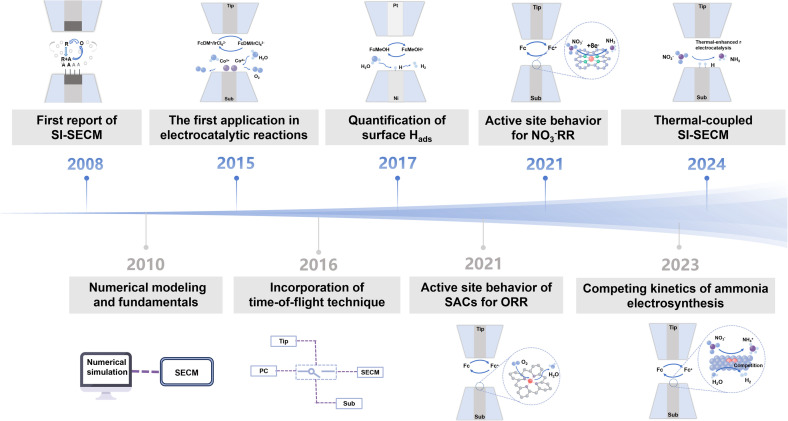
Development and historical evolution of SI-SECM.^14,17–24^

Subsequent technological developments have demonstrated a clear trend toward broader applications of SI-SECM. In 2017, Liang *et al.* applied the technique to study the HER and revealed the rate-determining nature of the Volmer step.^21^ In 2021, Jin *et al.* used SI-SECM in single-atom catalyst (SAC) research to elucidate the regulatory mechanism of Fe–N_4_ site spacing (<1.2 nm) on oxygen reduction reaction (ORR) activity.^22^ Between 2023 and 2024, the technique was further extended to complex reaction systems such as the nitrate reduction reaction (NO_3_^−^RR), enabling precise kinetic measurements of intermediates on Cu/Fe SAC surfaces and showcasing the potential for studying thermo-electrochemical multi-field coupling.^23–25^ This series of research advances highlights the unique strengths of SI-SECM in uncovering catalytic mechanisms (Fig. 1).

SI-SECM is a powerful characterization technique that has garnered significant attention for its ability to measure reactive species in electrocatalytic and photoelectrocatalytic reactions with high sensitivity and spatiotemporal resolution. It has been widely applied in the study of key processes such as the HER,^2–5,26–28^ oxygen evolution reaction (OER),^29–32^ ORR,^33,34^ CO_2_RR,^6,35^ nitrogen reduction reaction (NRR) and NO_3_^−^RR^23–25^ Beyond these applications, SI-SECM has demonstrated outstanding capabilities in characterizing advanced materials such as SACs, perovskite oxides, and layered double hydroxides (LDHs). It enables in-depth analysis of the dynamic behavior of active sites, the formation and transformation of intermediates, and the real-time monitoring of catalyst stability and activity, thereby greatly enhancing our understanding of interfacial reaction mechanisms.^14,22^ This technique not only accelerates progress in fundamental electrocatalysis research, but also provides critical insights for the rational design of efficient catalysts, making it highly significant for the advancement of sustainable energy technologies. Given the broad applicability and interdisciplinary significance of this technique, we believe this work will be of interest to researchers across various fields.

## Instrumentation and fundamentals

### SECM instrumentation and working mode

SECM consists of four electrodes: a tip electrode, a counter electrode, a reference electrode, and a substrate electrode. The tip electrode is mounted on a three-dimensional positioning system, allowing it to move precisely along the *X*, *Y*, and *Z* directions over the substrate. To achieve synchronized coordination between electrochemical measurements and positional control, the system employs an integrated control platform that combines tip positioning with electrochemical detection circuitry. This integration fundamentally avoids conflicts between separate modules.^36^ As a result, the sensitivity of SECM is determined by both the size of the tip electrode and the spatial resolution of the piezoelectric actuator used to move the tip.

Since the development of SECM, a variety of operational modes—such as the “feedback mode,” “generation–collection mode,” and “surface interrogation mode”—have provided diverse approaches for electrochemical microscale characterization.^37^ Among them, the feedback mode serves as the fundamental working mode. In this configuration, the tip current (*i*_T_) is determined by the diffusion rate of the redox species, and when the electrode radius is comparable to the diffusion layer thickness, a steady-state limiting current is achieved (*i*_∞_ = 4*nFDCa*). By analyzing the positive or negative feedback caused by conductive or insulating substrates, one can characterize both surface topography and reactivity. In contrast, the generation–collection mode relies on a redox cycle between the tip and substrate electrodes. It includes two complementary configurations: tip generation–substrate collection and substrate generation–tip collection. These modes are used for studying homogeneous reaction kinetics and probing the local activity of macroscopic electrodes, respectively.

The precise characterization of surface-adsorbed species—including reactants, products, and intermediates—is crucial for elucidating the mechanisms of heterogeneous electrocatalytic reactions.^38,39^ As an innovative transient mode, SI-SECM enables the dynamic tracking of surface-bound intermediates through an electrochemical titration strategy.^17^ In this technique, the target electrocatalyst serves as the substrate electrode, while a microscale tip probe generates redox mediators *in situ* to quantitatively titrate the surface-adsorbed species. By precisely controlling the type of mediator and the spatiotemporal parameters of the reaction, SI-SECM provides key insights into surface coverage, chemical potential, and reaction kinetics of intermediates. This offers a unique *in situ* characterization platform for advancing the mechanistic understanding of electrocatalytic processes.

The SI-SECM technique was initially applied primarily to classical surface systems with relatively low reactivity, such as gold and platinum oxides.^40,41^ While these early studies confirmed the reliability of the method, its limitations in temporal resolution became increasingly apparent as research extended to highly reactive surface-adsorbed species such as hydroxyl radicals^42^ and high-valent transition metals.^43,44^ This limitation primarily stems from the inherent switching delay of commercial potentiostats, which makes it difficult to accurately capture fast-reacting surface intermediates. Therefore, the development of new methods capable of precisely controlling the timing between species generation and detection has become essential for enhancing the time resolution of SI-SECM. In 2015, Ahn and Bard *et al.* addressed this challenge by introducing an external rapid switching device, enabling precise control over the delay time (*t*_delay_) between the generation of reactive species and their detection at the tip.^20^ This critical breakthrough allowed for accurate quantification of the reaction rates at highly active sites, paving the way for the reliable extraction of kinetic parameters and providing a powerful characterization tool for studying a wide range of highly reactive intermediates.

### Principles of SI-SECM experiments

**Fig. 2:**
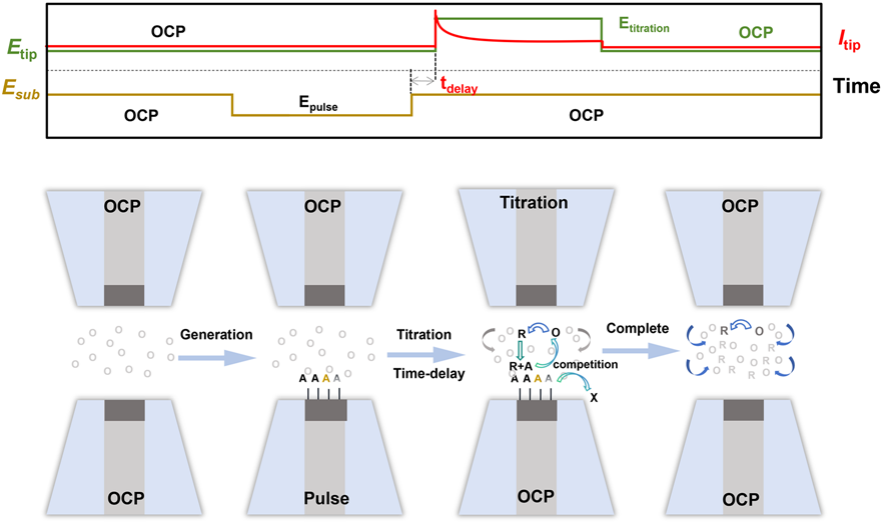
Schematic of the basic principle of the SI-SECM technique.

The operation of SI-SECM is based on the feedback mode of SECM and utilizes a unique open-circuit detection mechanism to enable the quantitative analysis of limited reactive species on the substrate surface. The core of this technique lies in the specific reaction between the redox pair (O/R) generated at the tip and the species adsorbed on the substrate, with real-time monitoring of the adsorbed amount through changes in the feedback current. Its innovation is reflected in the integration of both the generation and detection of reactive species, which not only simplifies the experimental setup but also significantly enhances detection sensitivity. The typical working mechanism of SI-SECM is shown in Fig. 2, comprising four key steps: (1) initial phase: both the tip and substrate remain in an open-circuit state, with no reactive intermediates generated on the substrate surface; (2) substrate potential pulse/scan phase: the substrate electrode operates at an oxidation potential, generating adsorbed reactive intermediate species A, while the detection tip remains in an open-circuit state; (3) titration phase: the substrate is switched to an open-circuit state, while the tip reduces O to R through potential scanning/pulses. R diffuses to the substrate surface, where it chemically reacts with A, accompanied by the regeneration of O. Importantly, during this interval, the intermediates A are not entirely stable—they are also consumed by the ongoing chemical reactions in the system. This introduces a competitive relationship between electrochemical and chemical steps for A. As a result, the time delay (*t*_delay_) between the generation of intermediates and the titration step has a pronounced effect on the titration signal: longer *t*_delay_ values lead to decreased surface populations of A due to spontaneous chemical consumption, which reduces the amount available to react with R. By recording titration curves at different *t*_delay_ values and analyzing their decay trends, the consumption rate of intermediates can be extracted, enabling estimation of the corresponding chemical rate constant. (4) Endpoint determination phase: after A is completely consumed, the system exhibits a characteristic negative feedback signal. This entire process, based on the cyclic conversion of the redox mediator O/R, allows for the *in situ* quantitative detection of adsorbed species A on the surface.

### Experimental conditions for SI-SECM

#### Tip and substrate size requirements

In SI-SECM experimental design, the microscale tip must be precisely aligned concentrically with the substrate electrode—typically a microdisk electrode matched to the tip size—and maintain a normalized distance of *L* = *d*/*a* ≤ 0.3. The precise matching of the probe and substrate electrode dimensions ensures that the current feedback can most accurately reflect the concentration changes of surface-adsorbed species, thereby enhancing the sensitivity and accuracy of the measurements. This close-spacing configuration offers two major advantages: (1) significantly enhanced collection efficiency^36^ and (2) improved contrast between positive and negative feedback signals. To ensure optimal test conditions, the substrate size must be strictly controlled. An excessively large substrate may lead to open-circuit positive feedback, caused by non-local mediator regeneration through lateral charge transfer pathways.^45–47^ Conversely, slightly reducing the substrate size can effectively suppress this effect, ensuring that mediator regeneration only occurs in the presence of active surface-adsorbed species—a critical condition for the quantitative analysis of adsorbates. This precise spatial configuration enables SI-SECM to achieve high-sensitivity detection of surface-adsorbed species.

#### Selection of redox mediators

In SI-SECM studies, the redox mediator (R) serves as a signal-transmitting carrier in the electrochemical process, and its selection is of critical importance. The typical reaction between the active intermediate (A) and the redox mediator (R) can be expressed as:R + A → O + P

An ideal mediator should meet the following criteria: (1) moderate solubility in the solvent system under investigation; (2) fast outer-sphere electron transfer kinetics; (3) good chemical stability; (4) an appropriate standard potential difference (Δ*E*^0^) relative to the target surface species; (5) negligible background interference on inert substrate electrodes.^36^ These properties collectively ensure high selectivity and accuracy in the detection of surface-bound intermediates. Table 1 below summarizes common redox couples for reference.

**Table 1:** Representative oxidation–reduction mediators used in aqueous solutions^44^

Redox half-reaction of mediator	Formal potential (V *vs.* NHE)	Applicable pH range	Redox half-reaction of mediator	Formal potential (V *vs.* NHE)	Applicable pH range
Ru(bpy)_3_^3+/2+^	1.27	∼pH 2–9	Fe(CN)_6_^3−/4−^	0.36	∼pH 3–10
Ru(phen)_3_^3+/2+^	1.22	∼pH 4–7	Co(bpy)_3_^3+/2+^	0.32	∼pH 3–9
Br_2_/Br^−^	1.09	∼pH 0–6	1,4-Benzoquinone/hydroquinone	0.28	∼pH 2–11
Fe(phen)_3_^3+/2+^	1.07	∼pH 3–5	*N*,*N*,*N*′,*N*′-tetramethyl-*p*-phenylenediamine (TMPD)	0.27	∼pH 3–10
Fe(bpy)_3_^3+/2+^	1.07	∼pH 4–7	Ru(en)_3_^3+/2+^	0.18	∼pH 2–9
IrCl_6_^2−/3−^	1.00	∼pH 0–5	1,2-Naphthoquinone/naphthohydroquinone	0.14	∼pH 2–10
Ru(bpy)_2_(NH_3_)_2_^3+/2+^	0.88	∼pH 5–7	FeEDTA^−/2-^	0.12	∼pH 3–10
Ru(CN)_6_^3−/4−^	0.86	∼pH 0–8	Ru(NH_3_)_6_^3+/2+^	0.05	∼pH 2–9
Os(bpy)_3_^3+/2+^	0.84	∼pH 4–7	Methylene blue	0.01	∼pH 4–10
Mo(CN)_8_^3−/4−^	0.77	∼pH 4–10	Co(en)_3_^3+/2+^	−0.22	∼pH 3–9
1,1-Dicarboxylic acid ferrocene	0.64	∼pH 3–10	Anthraquinone-2-sulfonate/anthrahydroquinone	−0.22	∼pH 4–9
Co(oxalate)_3_^3−/4−^	0.57	∼pH 4–8	Methyl viologen^2+/+^	−0.45	∼pH 6–9
W(CN)_8_^3−/4−^	0.49	∼pH 4–10	Co(sepulchrate)^3+/2+^	−0.54	∼pH 6–9
Ferrocene methanol^+/0^ (FcMeOH^+/0^)	0.44	∼pH 1–10	4,4^'^-Dimethyl-1,1′-trimethylene-2,2′-bipyridyl	−0.69	Not available
Co(phen)_3_^3+/2+^	0.38	∼pH 3–9	Fe(TEA)_2_^3+/2+^ (TEA = triethanolamine) (1M NaOH)	−0.82	∼pH 12–14
CoEDTA^−/2−^	0.38	∼pH 3–10	—	—	—

## Recommended practices

### Fabrication of ultramicroelectrodes (UMEs)

The conventional methods for preparing SECM tips are primarily based on the fabrication of disc-shaped metal nanoelectrodes (or nano-tips) using commercial CO_2_ laser pullers. The most common techniques for constructing UMEs include the glass encapsulation method and the epoxy resin encapsulation method.^48–55^

The basic steps of the glass encapsulation method include assembly, pre-pulling, sealing, and final pulling. First, a borosilicate glass capillary (*e.g.*, 1.0 mm outer diameter/0.2 mm inner diameter) is sealed at one end with a paraffin wax film. A ∼1 cm-long metal wire is then positioned coaxially within the capillary, and the assembly is placed in the groove of a CO_2_ laser puller. Next, a preliminary pull is applied to shape the electrode, followed by laser heating to achieve fused sealing between the metal wire and the glass tube. Finally, a second pulling step is performed to achieve the desired metal tip size. It is important to note that the program and parameter settings used during the pulling step should be adjusted based on laser conditions and the age of the instrument. Relevant parameters include heat (H), filament (F), velocity (V), delay (D), and pull (P).^36^ Throughout the entire process, it is critical to ensure that the platinum wire and glass tube are stretched synchronously to achieve optimal sealing quality.

The epoxy resin encapsulation method primarily involves three steps: pulling, assembly, and sealing. First, an empty glass tube is stretched to create a capillary for later use. Then, a ∼1 cm-long metal wire is soldered and fixed to a nickel–cadmium wire using conductive silver paste and inserted into the pulled glass capillary, ensuring that the front end of the metal wire is properly exposed. Next, epoxy resin is introduced into the tube using negative pressure from a vacuum pump, allowing it to fully fill the metal connection area. Finally, the assembly is left to stand vertically at room temperature until the epoxy cures, after which it is polished and ready for use. Compared to the traditional glass encapsulation method, this approach eliminates the need for pre-pulling the metal wire. By optimizing the encapsulation structure and using epoxy resin, the process is simplified while still ensuring reliable sealing and good mechanical strength of the encapsulated unit.

### Catalyst loading

In the process of detecting reactive intermediates or determining kinetic rate constants during electrocatalytic reactions using SI-SECM, the catalyst sample needs to be loaded onto the substrate electrode surface using specific methods. The current mainstream catalyst loading methods include etching and deposition techniques.^56–62^

The etching method is primarily used for the immobilization of powdered catalysts. First, controllable electrochemical etching is applied to the metal wire at the center of a microdisc electrode by applying an external voltage, creating micron-sized pore structures with specific dimensions. Subsequently, the powdered catalyst is uniformly loaded onto the modified ultramicroelectrode surface using a mechanical pressing technique, resulting in the preparation of the SI-SECM working electrode. Additionally, for larger electrodes (*e.g.*, 300 μm), the catalyst can be dispersed in a liquid medium and uniformly drop-cast onto the substrate surface. After drying, this forms a stable and active catalytic layer.

The deposition method is suitable for the immobilization of catalyst precursor solutions. By controlling the electrode potential, the active components in the precursor solution are selectively deposited onto the surface of the substrate electrode.

### Tip-substrate alignment

After pretreating the substrate and tip electrodes, the substrate is mounted in an SI-SECM electrochemical cell made of inert materials such as polycarbonate or polytetrafluoroethylene. The cell integrates ports for both reference and counter electrodes, forming a compact four-electrode system. It is then fixed onto a three-axis SECM positioning platform and leveled using a precision spirit level. The tip electrode is mounted onto the piezoelectric controller *via* a probe holder and coarsely aligned. Optical alignment and focus calibration are subsequently performed by imaging the substrate surface through a microscope system onto a computer interface, thereby establishing a spatial reference for subsequent measurements. This standardized setup minimizes the effects of mechanical vibration and positional errors.

In SI-SECM experiments, maintaining a normalized distance between the tip and substrate electrodes (*L* = *d*/*a* ≤ 0.3) is critical for acquiring reliable signals. Initially, the tip is coarsely positioned approximately 100 μm above the substrate. Electrolyte containing a redox mediator is then added to fully immerse the four-electrode system. Upon activating the potentiostat and SECM control software, the tip is driven downward along the *Z*-axis *via* piezoelectric control, while monitoring the feedback current in real time. When a distinct positive feedback signal appears and the current reaches 2–3 times the steady-state value, the tip achieves near-maximal collection efficiency for redox intermediates. A constant-height *X*–*Y* scan is then conducted to locate the optimal lateral position (*X*_0_, *Y*_0_) based on the peak feedback signal, completing precise three-dimensional positioning. The entire approach process is governed by a closed-loop system, ensuring high spatial resolution and reproducibility and providing a stable coordinate framework for high-precision electrochemical imaging.

### Potential screening in SI-SECM

In the SI-SECM experiment, the number of adsorbates at different potentials can be analyzed.^44^ For different catalytic systems, the potential corresponding to the maximum adsorbate quantity varies significantly, primarily depending on the intrinsic electrochemical activity of the catalytic material. The potential screening experiment follows a standardized procedure: First, the baseline is measured under open-circuit conditions (*i.e.*, zero bias) at the substrate electrode to obtain the background current response. Then, based on the characteristic redox potential range of the catalytic material, a step potential method is employed with 50 mV or 100 mV increments to systematically investigate the electrochemical response at different polarization potentials. By comparing the faradaic current responses at each potential, the characteristic potential that generates the maximum steady-state current response is identified as the optimal titration potential. This potential corresponds to the best electrochemical activation state of the catalyst's active sites, ensuring optimal signal response in subsequent time-delayed SI-SECM experiments. Specifically, when the redox mediator generated at the probe reacts with surface-bound intermediates at different substrate potentials, the recorded titration curves reflect not only the surface coverage of these intermediates but also their reaction rates. Intermediates with higher reaction rates produce curves characterized by a rapid rise and fast decay of current, resulting in a short overall response time. In contrast, slower-reacting intermediates exhibit a more gradual current increase, delayed decay, and prolonged signal duration.^63^ By acquiring titration curves over a range of substrate potentials and comparing their response features, it is possible to reveal differences in the reactivity of active sites under distinct electronic states or local environments. Furthermore, numerical simulations can be used to fit the titration curves precisely, allowing not only the determination of overall kinetic rates but also the identification of distinct types of active sites and the extraction of their individual kinetic parameters. This approach extends titration signals from a purely quantitative measurement to a comprehensive tool for correlating active site types with kinetic characteristics.

### Time-delay SI-SECM

**Fig. 3:**
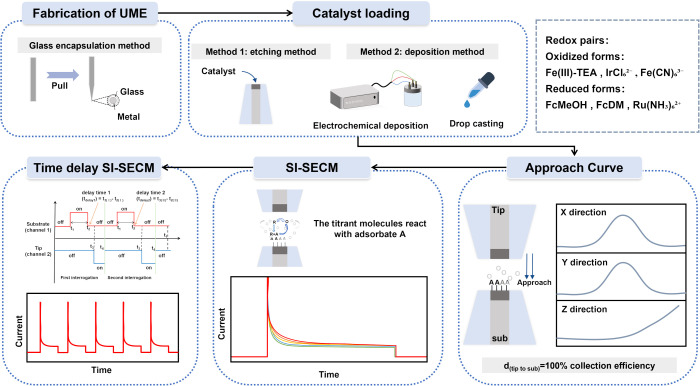
Schematic illustration of the typical experimental procedure for SI-SECM.

After selecting the optimal titration potential, delayed titration is performed at this potential. The core of time-delay SI-SECM lies in establishing a complete analytical chain from the raw signal to the reaction mechanism. Through preprocessing techniques, such as background subtraction, experimental noise can be effectively removed, and the true electrochemical response signal can be extracted. Time-delay SI-SECM involves placing a precisely controlled *t*_delay_ between the generation of the titrant and its reaction with the surface-active intermediate. By adjusting the time delay, the detection accuracy of short-lived intermediates can be significantly improved, thereby enabling a more accurate extraction of the kinetic constants. The titration current decay is then plotted as a function of *t*_delay_, which allows the determination of the kinetic rate constant for the intermediate under study. Similar micro-titration experiments can be conducted at different substrate potentials to quantitatively detect all relevant reaction intermediates, providing a comprehensive understanding of the reaction mechanism. The recommended standard procedure for SI-SECM is shown in Fig. 3.

### Data analysis

In the SI-SECM experiment, the titration curves are collected at different *t*_delay_ to quantitatively analyze the concentration of reactive intermediates ([A*]) and determine the reaction kinetics parameters. First, the titration *i*_tip_–*t* curves are integrated and the background response under open-circuit conditions is subtracted to obtain the charge transferred (*Q*). This value is then normalized by the electrochemically active surface area (ECSA) to correct for the effects of uneven distribution of active sites on the substrate surface. The ECSA is typically measured using the double-layer capacitance method, and its calculation formula is as follows:*A*_ECSA_ = *C*_DL_/*C*_DL,metallic o*x*ide_*C*_DL_ represents the double-layer capacitance of the catalyst measured using CV at different scan rates; *C*_DL,metallic o*x*ide_ indicates that the ideal specific capacitance value for a metal oxide is 60 μF cm^−2^.

Based on Faraday's law, the concentration of active intermediates can be calculated using the following formula:[A*] = *Q*/*nFA*_ECSA_In the equation, *F* is the Faraday constant (96 485 C mol^−1^) and *n* represents the net number of electrons transferred in the target half-reaction. To obtain the reaction rate constant (*k*′), a first-order reaction is used as an example: the natural logarithm of the intermediate concentration, ln[A*], at each delay time is plotted, and the slope of the resulting linear fit corresponds to the apparent rate constant of the reaction. This method enables the accurate quantification of electrochemically active intermediates and reliable analysis of reaction kinetics by simultaneously analyzing SI-SECM signals and ECSA corrections. It is particularly suitable for studying complex electrochemical reaction systems involving short-lived intermediates.

### Recent progress of SI-SECM for studying electrocatalysis

#### HER

**Fig. 4:**
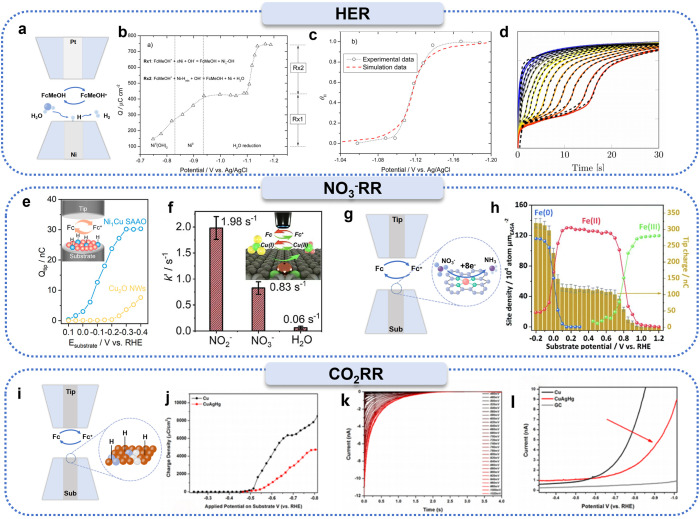
Applications of SI-SECM in studying surface active hydrogen species induced electroreduction reactions. (a) Schematic illustration of hydrogen adsorption on a nickel electrode surface using SI-SECM. (b) Charge density as a function of applied potential. (c) Hydrogen adsorption coverage *versus* applied potential. Reproduced with permission from ref. 21, American Chemical Society, 2017. (d) Overlay of experimentally measured current responses at a glassy carbon tip electrode with simulated current responses for three distinct adsorption sites. Reproduced with permission from ref. 62, Wiley-VCH, 2020. (e) Tip titration charge density *versus* potential for quantitative determination of surface-active hydrogen species on Cu_2_O nanowires and Ni_1_Cu single-atom alloy oxide catalysts. Reproduced with permission from ref. 25, American Chemical Society, 2024. (f) Kinetic rate constants for NO_2_^−^, NO_3_^−^, and H_2_O adsorption on copper single-atom grids measured by SI-SECM. Reproduced with permission from ref. 24, American Chemical Society, 2023. (g) Schematic of the SI-SECM setup for iron-site titration experiments. (h) Electrochemically active surface area-normalized active site density and corresponding titration charge as a function of potential. Reproduced with permission from ref. 23, Royal Society of Chemistry 2021. (i) Experimental design for detecting active hydrogen species during CO_2_ reduction using SI-SECM. (j) Redox titration curves of surface-active hydrogen on copper and Cu_10_Ag_14_Hg_0.6_ catalysts. (k) Chronoamperometric responses and derived charge density plots for surface-active hydrogen titration on Cu_10_Ag_14_Hg_0.6_ electrodes at various applied potentials. (l) Linear sweep voltammograms of hydrogen evolution for catalysts deposited on carbon ultramicroelectrodes in 0.1 M K_2_SO_4_ solution. Reproduced with permission from ref. 64, American Chemical Society, 2020.

Since the advent of SECM, this technique has been successfully applied in the study of key electrocatalytic systems such as the HER, NO_3_^−^RR, CO_2_RR, OER, ORR, and NRR. Notably, the mechanistic studies of the HER, NO_3_^−^RR, and CO_2_RR all involve the generation and transformation of active hydrogen intermediates (H_ads_). The emerging SI-SECM technique, with its unique high sensitivity, provides a powerful tool for real-time monitoring of these transient active hydrogen species. In this research field, Liang *et al.* developed the SI-SECM technique, using FcMeOH as a redox mediator, a Ni electrode as the substrate electrode, and a Pt UME as the probe. By measuring the current response of the Pt tip electrode, they were the first to establish a quantitative relationship between the hydrogen adsorption coverage on the Ni electrode surface and the applied potential (Fig. 4a–c).^21^ Their findings indicated that within a specific potential window, the Volmer step (electrochemical hydrogen adsorption step) exhibited distinct kinetic limitations, which was identified as the rate-determining step of the catalytic system. This pioneering work provides an important methodological reference for subsequent studies on electrocatalytic reaction mechanisms.

In addition, Leonard *et al.* combined the SI-SECM technique with finite element numerical simulations to study the formation of surface-adsorbed intermediates and their potential dependence during the HER on polycrystalline platinum in alkaline media (Fig. 4d).^62^ By comparing experimental and simulation data, they accurately identified and quantified three distinct types of adsorption intermediates formed on the Pt surface during the HER in alkaline media. The characteristics of these intermediates and their impact on HER kinetics revealed the main reason for the slow HER kinetics in alkaline media, namely the slow rate of the hydrogen atom-water molecule reaction to form hydrogen gas and the surface saturation phenomenon. These findings provide important theoretical insights for the design of more efficient HER electrocatalysts.

#### NO_3_^−^RR

In the past two years, the application of SI-SECM has expanded to more complex systems such as the NO_3_^−^RR. Liu *et al.* used FcMeOH as a redox mediator, loading Ni-site-modified Cu single-atom alloy oxide nanowires (Ni_1_Cu SAAO NWs) and copper(i) oxide nanowires (Cu_2_O NWs) onto UMEs as substrate electrodes. By integrating the steady-state current response curves, they successfully compared the concentration of H_ads_ on the surfaces of Ni_1_Cu SAAO NWs and Cu_2_O NWs (Fig. 4e).^25^ The study showed that the H_ads_ concentration on the Ni_1_Cu SAAO catalyst increased with a negative shift in potential, reaching four times that on Cu_2_O NWs at −0.3 V *vs.* RHE, and that the introduction of Ni single atoms significantly promoted water dissociation.

In addition to detecting the coverage of active intermediates on the catalyst surface, SI-SECM can also obtain kinetic parameters of interfacial electron transfer with nanometer spatial resolution and millisecond time resolution. Li *et al.* used FcMeOH as a redox mediator, loading copper single-atom gel (Cu SAGs) electrocatalysts onto UMEs as substrate electrodes. They studied the dynamic accumulation and transformation behavior of NO_2_^−^ intermediates during the NO_3_^−^RR on Cu SAGs (Fig. 4f).^24^ Real-time monitoring with SI-SECM revealed that the adsorption rate constant of NO_2_^−^ (1.98 s^−1^) was significantly higher than that of NO_3_^−^ (0.83 s^−1^) and water molecules (0.06 s^−1^), indicating that the rapid desorption of NO_2_^−^ is one of the rate-limiting steps in the NO_3_^−^RR. Additionally, Cu SAGs effectively suppress the interference from the competing HER. Furthermore, the team employed SI-SECM to compare the NO_2_^−^ reduction reaction (NO_2_^−^RR) mechanism of iron-based single-atom catalysts under conditions with and without NO_3_^−^ (Fig. 4g–h).^23^ By integrating the chronoamperometric curves (*i*–*t*), they observed two distinct current jumps and, combined with potential step experiments, proposed a catalytic mechanism in which the Fe active site undergoes oxidation state transitions (Fe^2+^/Fe^3+^) during the reaction.

#### CO_2_RR

In the study of the CO_2_RR, a key scientific challenge is to control the reaction pathway to achieve high selectivity for target fuel products while effectively suppressing the competing HER. Kim *et al.* innovatively employed a CuAgHg ternary metal thin-film catalyst system, which was loaded onto a UME as a substrate electrode, and used FcMeOH as a redox mediator. They successfully performed *in situ* electrochemical titration analysis of H_ads_ on the catalyst surface (Fig. 4i–l).^64^ The results indicated that the H_ads_ concentration on the CuAgHg catalyst surface was significantly lower than that on pure copper, suggesting that the introduction of mercury notably reduced the H_ads_ concentration on the catalyst surface. From a kinetic perspective, the study elucidated the intrinsic mechanism by which this suppression of the HER occurs, providing an important theoretical basis for the design of efficient CO_2_RR catalysts.

#### OER

**Fig. 5:**
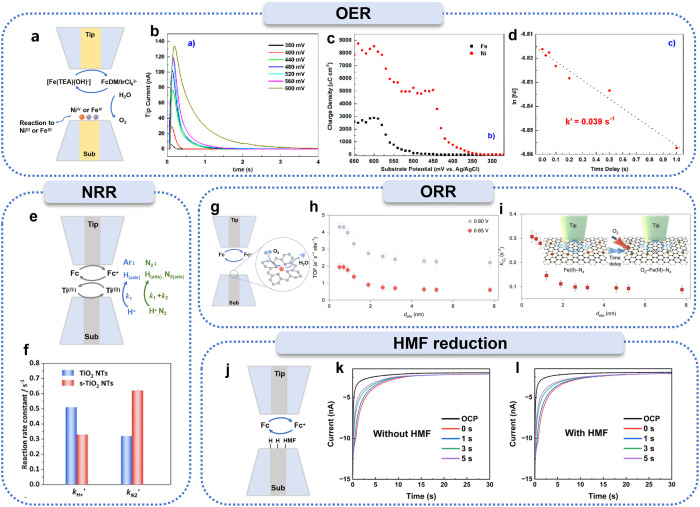
Applications of SI-SECM for investigating various active intermediates. (a) Schematic illustration of SI-SECM for OER studies. (b) Chronoamperometric traces for titrating Ni(OH)_2_ at different substrate potentials (*E*_subs_). (c) Redox titration curves obtained from Ni(OH)_2_ (red) and FeOOH (black) electrodes. (d) Time-delayed titration of Ni(OH)_2_. Reproduced with permission from ref. 91, American Chemical Society, 2015. (e) Schematic representation of SI-SECM for NRR studies. (f) Adsorption rate constants of H^+^ and N_2_ at Ti sites on TiO_2_ nanotubes and s-TiO_2_ nanotubes. Reproduced with permission from ref. 60, Wiley-VCH, 2020. (g) Schematic diagram of SI-SECM for ORR investigations. (h and i) Quantification of active Fe sites by SI-SECM at identical potentials, showing time-delayed titration curves for O_2_ binding reaction rate constants at Fe(iii)–N_4_ sites. Reproduced with permission from ref. 22, Springer Nature, 2021. (j) Experimental setup for SI-SECM studies of the electrochemical hydrogenation of HMF. (k and l) Current responses of adsorbed intermediates on Ag electrode surfaces measured by SI-SECM at various applied potentials. Reproduced with permission from ref. 92, Royal Society of Chemistry 2025.

Beyond hydrogen-related processes, the application of SI-SECM has steadily expanded to other important electrocatalytic reactions, such as the OER, NRR, and ORR. The OER is a key process in energy conversion, and many catalyst candidates are actively being investigated. Oxides of iridium,^65–76^ cobalt,^77–83^ and nickel^84–87^ are commonly studied. Currently, the application of SI-SECM in OER electrocatalyst characterization has expanded to the identification of multiple types of catalytically active sites in mixed metal oxide systems.^88^ Nickel–iron oxides, as classic OER catalysts, have been extensively studied since the late 1980s.^89,90^ Ahn *et al.* systematically controlled the nickel-to-iron ratio to prepare a series of mixed oxide catalysts (Fig. 5a–d),^91^ which were loaded onto substrate electrodes. Using iron complexes of triethanolamine as redox mediators and measuring the tip current changes, they successfully confirmed—*via* time-dependent titration methods—that iron sites dispersed in the NiOOH continuous matrix are the primary contributors to high OER activity. The study also revealed that the surfaces of Ni(OH)_2_, FeOOH, and Ni_1−*x*_Fe_*x*_OOH electrodes exhibit high-density catalytically active sites (approximately 300 atoms per nm). Furthermore, they found that the OER kinetics of Ni^IV^ were poor (*k'* = 0.04 s^−1^), while “fast” (*k'* = 1.70 s^−1^) and “slow” OER active sites were present on FeOOH and Ni_1−*x*_Fe_*x*_OOH (*x* < 0.25) electrodes, with the proportion of “fast” sites correlating with the iron atomic content. This suggests that these “fast” sites may be isolated iron atoms dispersed within the NiOOH matrix. This discovery provides direct experimental evidence for understanding the structure–activity relationship of nickel-iron oxide catalysts.

#### NRR

The NRR for ammonia synthesis is an important method for simulating the natural nitrogen fixation process, and it holds irreplaceable strategic value in industrial applications.^90,93,94^ Li *et al.* systematically studied the mechanism by which lattice strain affects the electrocatalytic NRR performance by constructing geometrically optimized TiO_2_ nanoreactors (Fig. 5e–f).^60^ The results indicated that tensile strain on the surface significantly enhanced the catalytic activity of TiO_2_. Using the SI-SECM technique with FcMeOH as a redox mediator, the catalysts were loaded onto substrate electrodes, and SI-SECM experiments revealed that Ti^3+^ species are the main active sites for both the NRR and the HER. Through quantitative analysis, the N_2_ adsorption rate constant 
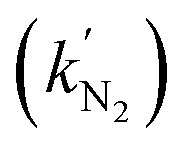
 for s-TiO_2_ NTs was found to be 0.62 s^−1^, nearly double that of TiO_2_ NTs (0.32 s^−1^), indicating that strain significantly enhanced the adsorption capacity of Ti^3+^ sites for N_2_ and improved NRR selectivity. Additionally, by modulating the electronic structure, the Ti^3+^ active sites were stabilized, which significantly improved the NRR kinetics.

#### ORR

The control of site density in SACs has been proven to be an effective strategy for optimizing electrocatalytic performance,^95–97^ such as for the ORR. However, the intrinsic catalytic behavior of individual active sites and their synergistic interaction mechanisms remain poorly understood. Jin *et al.* used SI-SECM to reveal the mechanism of enhanced ORR activity in Fe–N_4_ SACs at the sub-nanometer scale (Fig. 5g–i).^22^ Analysis of the active site density measured by SI-SECM showed that the atomic utilization efficiency of iron atoms in SACs decreases with increasing Fe content, and the active site density is closely related to the Fe atomic spacing. Using the SI-SECM time-delay titration technique, it was found that the reaction rate constant (*k'*) of Fe^II^ active sites with oxygen significantly increased when the Fe atomic spacing was less than 1.2 nm. The strong interactions between adjacent Fe–N_4_ sites altered the electronic structure, leading to an increase in intrinsic ORR activity. This finding provides an important theoretical foundation for understanding the structure–activity relationship of SACs.

#### HMF reduction

Recently, the SI-SECM technique has been innovatively applied to the study of electrocatalytic organic small molecule reaction mechanisms. Choi *et al.* applied SI-SECM to investigate the electrochemical hydrogenation of 5-hydroxymethylfurfural (HMF), not only achieving *in situ* quantitative analysis of the adsorbed intermediates during the reduction process of HMF but also revealing the competitive mechanism between HMF reduction and the HER (Fig. 5j–l).^92^ By measuring the current response of adsorbed intermediates on the surface of an Ag electrode using SI-SECM, they found that as the potential became more negative, the concentration of adsorbed intermediates increased, leading to an increase in the titration current. However, when the potential exceeded −1.3 V, the current response began to decrease, indicating an increased consumption rate of the adsorbed intermediates. This trend revealed that within the potential range of −1.3 V to −1.5 V, the electrochemical hydrogenation mechanism contributed most significantly to the formation of 2,5-bis(hydroxymethyl)furan (BHMF), while more negative potentials promoted the HER, causing more adsorbed intermediates to be used for the HER, thus reducing the yield of BHMF. This study not only provides a new perspective on the electrochemical transformation of HMF but also opens up new pathways for investigating the mechanisms of electrocatalytic organic small molecule reactions.

Table 2 below summarizes the applications of the SI-SECM technique in various catalytic reactions to date, with a focus on the electrode materials used in SI-SECM experiments, the catalytic reactions involved, the redox mediators employed, and the types of reactive intermediates detected.

**Table 2:** Summary of reported applications of SI-SECM in different catalytic reactions

Electrocatalytic reactions	Electrode materials	Redox mediators	Adsorbed intermediates	Ref.
HER	Au UME	1,1′-Ferrocenedimethanol (FcDM)	H_ads_	2
	Tip: GC UME	FcMeOH	*H*_UPD_, *H*_OPD_	62
	Sub: Pt UME			
	Tip: Pt UME	FcMeOH	H_ads_	21
	Sub: Ni UME			
	Pt UME	FcMeOH	H_ads_	98
OER	Tip: Au UME	Methyl viologen^+^ [Ru(NH_3_)_6_]^2+^	PtO_*x*_	18
	Sub: Pt UME			
		Fe(ii)[EDTA]^2−^		
		Fe(CN)_6_^4−^		
	Tip: Au UME	Ru(phen)_3_^2+^	Mn^3+^, Mn^4+^, Mn^5+^	58
	Sub: C UME	IrCl_6_^2−^		
	Tip: Au UME	IrCl_6_^2−^	˙OH_(ads)_	99
	Sub: TiO_2_			
	Tip: GC UME	Fe(iii)-TEA	Ni^IV^, Fe^IV^	89
	Sub: Ni_0.8_: Fe_0.6_ electrode on FTO			
	Tip: Au UME	Fe(CN)_6_^3−^	Cu^III^	100
	Sub: CuO Au UME			
	Tip: Au UME	[Fe(MeOH)]^+^	Fe^4+^	101
	Sub: α-Fe_2_O_3_ on FTO			
	Tip: Au UME	Fe(CN)_6_^3−^	˙OH_(ads)_	102
	Sub: Ti–Fe_2_O_3_ on FTO			
	Tip: Au UME	Fe(iii)-TEA	Co^III^/Co^IV^	103
	Sub: Co_3_O_4_			
	Tip: Au UME	FcDM^+^/IrCl_6_^2−^	Co^III^/Co^IV^	19
	Sub: CoPi-Au UME			
	Au UME	[Fe(TEA)(OH)]^−^	Ni^IV^, Fe^IV^	91
OER	Tip: Pt UME	FcMeOH	Fe^II^, Co^II^	104
ORR	Sub: CoFe-PPy			
ORR	Tip: Pt UME	FcMeOH	Fe^II^, Fe^III^	22
	Sub: FeN_4_–Pt UME			
	Pt UME	FcMeOH	Cu^I^	105
NO_3_^−^RR	Tip: Pt UME	FcMeOH	NO_3_^−^, NO_2_^−^	56
	Sub: Cu SAA			
	Tip: Pt UME	FcMeOH	NO_3_^−^, NO_2_^−^, H_2_O	24
	Sub: Cu SAGs			
NO_3_^−^RR	Tip: Pt UME	FcMeOH	H_ads_	25
	Sub: Ni_1_Cu SAAO			
	Tip: Au UME	IrCl_6_^2−^	˙Cl, ˙OH	106
	Sub: Ti–C UME			
NitRR	Tip: Pt UME	FcMeOH	H_ads_	107
	Sub: Cu (LDHs)			
NRR	Tip: Pt UME	FcMeOH	Ti^3+^	60
	Sub: s-TiO_2_ NTs			
CO_2_RR	Tip: Pt UME	FcMeOH	H_ads_	64
	Sub: CuAgHg			
CO oxidation	Pt UME	Br^−^	CO_ads_	108
HMF reduction	Tip: Ag UME	FcMeOH	HMF_ads_	92
	Sub: C UME		H_ads_	

## Conclusion and Outlook

**Fig. 6:**
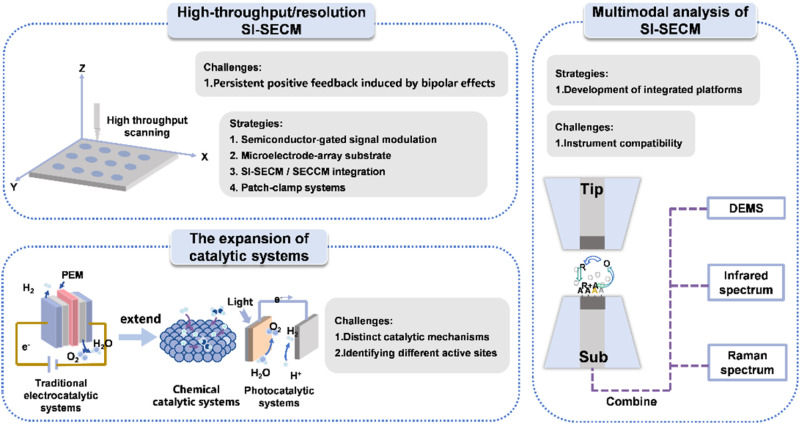
Schematic illustration of future directions for the development of SI-SECM.

The SI-SECM technique has demonstrated significant methodological value in the field of electrocatalysis research, with pioneering contributions in the following areas: (1) optimization of product selectivity through precise control of the electrocatalyst's stoichiometric composition; (2) elucidation of complex electrocatalytic reaction mechanisms; and (3) *in situ* quantitative analysis of reaction intermediate concentrations. With advances in nanoelectrode fabrication techniques, this approach is expected to become a powerful characterization tool for key electrocatalytic reactions in the processes of renewable energy storage, conversion, and utilization.^14,58,109,110^ In this regard, we predict that the SI-SECM technique will evolve towards “high throughput,” “expansion of catalytic systems,” and “multimodal analysis technologies” in the future, as shown in Fig. 6.

### High-throughput/resolution SI-SECM

Achieving both high-throughput and high-resolution measurements remains a central challenge in the application of SI-SECM. In the context of high-throughput screening, a major limitation arises from the requirement to precisely match the tip size to the substrate size to avoid spontaneous positive feedback at the open-circuit potential of conductive substrates. This constraint restricts rapid, large-area screening and complicates the interpretation of transient titration current signals. Two strategies can address this limitation: (1) applying a semiconductor gate-control strategy to modulate the conductive properties of the substrate *via* an external bias. This approach can suppress spontaneous positive feedback under open-circuit conditions without strict constraints on tip-substrate size matching, enabling accurate signal interpretation even when the size of the substrate is much larger than that of the probe; (2) developing microarray chip substrates based on integrated circuits. Coupled with circuit control, the electrochemical reaction of each channel can be individually controlled and measured, thereby enabling a universal high-throughput screening method.

For high-resolution measurements, both spatial and temporal dimensions must be considered. Regarding spatial resolution, micro- and nanoscale electrodes on conductive substrates are prone to positive feedback interference induced by bipolar effects, which is particularly pronounced for nanoelectrodes where the current signal is typically in the pA range. This overlap between positive feedback and titration signals complicates data interpretation. To overcome this, SI-SECM can be integrated with scanning electrochemical cell microscopy (SECCM), and a dual-channel probe can be developed that functions as both a nano-pipette and a nanoelectrode.^111,112^ This configuration enables the formation of a confined microdroplet that locally controls the conductive area of the substrate, thereby suppressing background positive feedback and improving spatial resolution. Temporal resolution is currently limited by the bandwidth of commercial potentiostats, which restricts high-frequency sampling while maintaining high current sensitivity and thus hinders accurate analysis of sub-millisecond reaction dynamics. High-time-resolution techniques from other single-entity electrochemical methods, such as patch-clamp amplifiers, offer higher sampling rates and faster signal response. Integrating such systems with SI-SECM could enable transient process analysis at the microsecond scale, providing detailed kinetic insights into ultrafast reactions.

### Expansion of catalytic systems

As an emerging *in situ* characterization method, the SI-SECM technique is expected to expand its application from traditional electrocatalysis systems to a broader range of catalytic fields. This development will mainly advance along three key directions: first, by coupling SI-SECM with multiple physical fields (*e.g.*, thermal, electric, and magnetic fields), a novel paradigm for catalytic research can be established. Second, the application potential of this technology in chemical catalysis, biocatalysis, and photocatalysis remains to be fully explored. Achieving these breakthroughs will require overcoming several key technical challenges, including distinguishing catalytic mechanisms, identifying different active sites, developing signal detection methods, addressing variations in reaction conditions, and ensuring technological adaptability.

### Multimodal analysis

Finally, we propose the systematic integration of the SI-SECM technique with various *in situ* spectroscopic characterization techniques, including but not limited to *in situ* Raman spectroscopy, *in situ* infrared spectroscopy, and mass spectrometry. Although SI-SECM offers excellent spatiotemporal resolution, it alone cannot resolve the electronic states and energy level information of reaction intermediates, making it challenging to distinguish chemical species or detect gaseous products, as both energy and mass resolution are relatively low. Combining SI-SECM with *in situ* spectroscopic techniques in a multimodal approach can address these limitations and enable comprehensive characterization of electrochemical interfaces. This multimodal strategy will lead to several key breakthroughs: (1) the establishment of an integrated characterization platform for (photo)electrocatalytic processes, allowing for the simultaneous acquisition of electrochemical signals and molecular structural information; (2) significant enhancement of the system's energy and mass resolution; and (3) a deeper understanding of critical scientific issues in catalytic reaction mechanisms, such as the dynamic evolution of active sites, charge transfer mechanisms at solid–liquid interfaces, and the real-time generation and consumption dynamics of reaction intermediates. This advanced multiscale characterization method will provide a novel research perspective for understanding complex catalytic systems and promote a paradigm shift in catalysis science from macro-performance studies to micro-mechanism analysis. It is important to note that significant technical challenges remain in coupling spectroscopic techniques within such tip-substrate gaps for quantitative analysis.

In summary, SI-SECM, as an emerging *in situ* electrochemical technique, has demonstrated unique advantages in the field of electrocatalysis. With continued development in high-throughput screening, system integration, and multi-field coupling, SI-SECM is poised to further advance mechanistic studies and the rational design of next-generation electrocatalysts. These advancements will offer powerful methodological support for addressing urgent global challenges in energy and environmental sustainability.

## Author contributions

Writing – original draft, R. Y.; visualization, W. Y. and L. Y.; writing – review & editing, Z. J. and M. Z.; funding acquisition, Z. J. and P. Li.; supervision, Z. J. and M. Z.

## Conflicts of interest

There are no conflicts to declare.

## Data Availability

No primary research results, software or code have been included and no new data were generated or analysed as part of this review.
